# Neurocognitive Performance in Depressed Patients with low-grade inflammation and somatic symptoms

**DOI:** 10.1016/j.bbih.2021.100409

**Published:** 2021-12-24

**Authors:** Muzaffer Kaser, Éimear M. Foley, Golam M. Khandaker

**Affiliations:** a Department of Psychiatry, University of Cambridge, Cambridge, UK; b Cambridgeshire and Peterborough NHS Foundation Trust, Cambridge, UK; c MRC Integrative Epidemiology Unit, Population Health Sciences, Bristol Medical School, University of Bristol, Bristol, UK; d Centre for Academic Mental Health, Population Health Sciences, Bristol Medical School, University of Bristol, Bristol, UK; e Avon and Wiltshire Mental Health Partnership NHS Trust, Bristol, UK

**Keywords:** Depression, Cognition, Inflammation, Reaction time, Neurocognitive, C-reactive protein

## Abstract

**Background:**

The link between inflammation and depression has been investigated extensively. Cognitive dysfunction in depression is an unmet treatment need. A better understanding of possible links between inflammation and cognition in people with depression may help to identify new treatment targets.

**Methods:**

We report findings from a study comparing a range of cognitive functions between 80 depressed patients with (C-reactive protein ≥3 ​mg/L; n ​= ​37) and without (CRP<3 ​mg/L; n ​= ​43) evidence of inflammation. All participants met the International Classification of Diseases 10th Revision criteria for current depressive episode and had somatic symptoms of depression. All participants completed cognitive testing and clinical assessment and were screened for acute infection.

**Results:**

Patients with evidence of inflammation, compared to those without, had slower psychomotor speed as measured by symbol coding task (mean difference ​= ​0.06, 95% CI ​= ​0.003–0.11) and slower reaction time, as measured by a simple movement time task (mean difference ​= ​26.56, 95% CI ​= ​-48.92 to −4.20). These effects were fully explained after controlling for age, sex, and body mass index. Measures of emotional processing, memory, and planning were comparable between two groups.

**Conclusions:**

Certain cognitive domains, particularly processing speed and reaction time may be more affected in depressed patients with evidence of low-grade inflammation and somatic symptoms. Further studies with larger samples are required for a clearer understanding of the association between inflammation and cognitive dysfunction in depression.

## Highlights

• Depressed patients with evidence of inflammation (CRP >3 ​mg/L) showed poorer neurocognitive test performance in psychomotor speed and reaction time.
• Neurocognitive. performance difference between patients with and without evidence of inflammation was no longer significant after controlling for age, sex, and BMI.
• The impact of depression severity and fatigue differed across cognitive domains; reaction time was affected more by fatigue scores while psychomotor speed was affected more by depressive symptoms.
• Stratification of patient samples with depression according to inflammation status may provide insights into cognitive dysfunction in depression.

## 1 Introduction

Over the last few decades, we have seen accumulation of compelling evidence on the link between inflammation and depression (Raison et al., 2006; Dantzer et al., 2008; Khandaker et al., 2017). Population-based studies reported association between inflammatory markers like C-reactive protein (CRP) and a range of depressive symptoms including fatigue, sleep and appetite disturbance (Jokela et al., 2016; Chu et al., 2019). Patients with depression show higher levels of peripheral inflammation markers such as C-reactive protein (CRP) (Osimo et al., 2019), and cytokines (Haapakoski et al., 2015) compared with controls. Increased levels of tumor necrosis factor-alpha (TNF-α) and interleukin-6 (IL-6) in cerebrospinal fluid and brain parenchyma were detected in patients with depression (Enache et al., 2019). Population-based prospective studies and genetic Mendelian randomization studies provided evidence to suggest a potential causal role of inflammation in depression that is not explained by reverse causality of residual confounding (Khandaker et al. 2014, 2020; Milaneschi et al., 2021; Ye et al., 2021). Other Mendelian randomization studies, however, did not confirm a direct relationship between CRP levels and depressive symptoms (Kappelmann et al., 2021; Perry et al., 2021). Inflammatory changes are associated with altered functional connectivity and enhanced responses to emotional stimuli in brain imaging studies (Kraynak et al., 2018). For instance, experimentally induced inflammation with typhoid vaccine induced low mood that was reflected in changes in critical brain networks implicated in emotion processing (Harrison et al., 2009). The link between immune response, genetic variations and vulnerability to depression is another important contributor to pathophysiology. Certain immune genes were implicated in depression, but the interaction between stress, environmental factors and physical health conditions needs further scrutiny (Barnes et al., 2017). Treatment studies also provided evidence for antidepressant effect of anti-inflammatory treatment in patients with chronic inflammatory disease (Kappelman et al., 2018; Jones et al., 2020).

Cognitive dysfunction is a key feature of depression and an unmet clinical need. Patients with depression report widespread difficulties in concentration, attention, and/or memory in addition to widely observed problems of anhedonia and vegetative symptoms (Roiser et al., 2012). Cognitive dysfunction in depression is associated with poorer clinical outcomes, impaired functioning (Baune et al., 2010), and increased risk for relapse (Majer et al., 2004). A significant group of patients with depression report persistent cognitive problems despite improvements in other symptoms of depression (Conradi et al., 2011). Emerging evidence from epidemiological and experimental studies points to a role of inflammation in cognitive dysfunction. Population-level studies show an association between poorer cognitive performance and IL-6 (Marsland et al., 2006) or CRP (Gimeno et al., 2008) levels. Higher IL-6 levels have been linked to reduced hippocampal volumes in middle-aged healthy volunteers (Marsland et al., 2008). Interventions that alter inflammation provide further evidence for this link between inflammation and cognitive function. For instance, treatment with interferon-gamma (a pro-inflammatory agent) has been shown to lead to slowing in psychomotor speed (Majer et al., 2008). Experimentally induced inflammation with typhoid vaccination has been shown to lead to poorer performance in a reaction time task which was associated with high IL-6 levels and substantia nigra activation (Brydon et al., 2008). Typhoid vaccine induced inflammation has been reported to induce low mood that was associated with changes in critical brain networks implicated in emotion processing (Harrison et al., 2009). A meta-analysis of functional neuroimaging studies and peripheral inflammation highlighted a role for limbic and basal ganglia circuits including amygdala, hippocampus, and striatum (Kraynak et al., 2018). It is suggested that the brain circuits associated with peripheral inflammation are also implicated in viscerosensory processes connecting brain and body. Those brain circuits are predominantly linked to psychomotor speed and emotional processing (Alvarez et al., 2020). Bottom-up information processing and interaction between different levels of cognitive functions in the context of depression were formulated in a cognitive neuropsychological model (Roiser et al., 2012).

In a recent literature review of cognitive symptoms in depression and inflammation, most studies reported no association, particularly after adjustment of vegetative symptoms (Majd et al., 2020). It should be noted that most of the studies investigating cognitive symptoms did not include objective neuropsychological measures and, therefore the results should be interpreted with caution. Among those that used neurocognitive tests (Chang et al., 2012; Krogh et al., 2014), the results on the relationship between cognitive function and inflammation in depression were mixed. Some studies reported an association between specific inflammatory markers and poorer cognitive performance in domains such as verbal memory (Grassi-Oliveira et al., 2011), reaction time (Goldsmith et al., 2016), and psychomotor speed (Krogh et al., 2014) while others reported no association (Chang et al., 2012). The direction of association, affected domains, and implicated inflammatory markers varied extensively. There is a need to use comprehensive cognitive tests with high reliability to document the association (or lack thereof) between inflammatory markers and cognition in depressed patients. Evidence from rodent studies and human neuroimaging studies point towards certain brain structures/circuits that are more sensitive to the effects of peripheral inflammation (Harrison, 2016). These include changes in ventral striatum (reward processing), hippocampus (memory), insula, cingulate and amygdala (multiple cognitive-emotional processes). It is key to investigate the neurocognitive performance associated with functioning of abovementioned brain circuits.

Neurocognitive tasks should be selected to tap into the cognitive domains that are widely affected in depression and that are candidate treatment targets. It is key to use tests that are well-established in terms of translational utility, reliability and their sensitivity to pharmacological manipulation. Distinct domains may still have shared underlying mechanisms. Yet, specific domains correspond to established brain circuits – and therefore their links to systemic inflammation – that can provide directions for therapeutic potential of anti-inflammatory drugs. Previous studies used one or two cognitive tests that limits the understanding of inflammation on different domains. Experimental studies suggested a closer link between peripheral inflammation and emotional/limbic processes (Harrison et al., 2016) that are also defined as lower order cognitive processes. Higher order cognitive functions are defined as faculties (e.g. planning) that are mediated by neural circuits higher up in cortical hierarchy (Disner et al., 2011; Roiser et al., 2012). To investigate the association between different levels of cognitive functions and inflammation, we conducted an exploratory study comparing a range of cognitive functions between depressed patients with and without evidence of inflammation. We hypothesized that lower order cognitive functions including psychomotor speed, reaction time, and emotional processing would be associated with CRP levels and the patients with higher CRP would have poorer performance on those tasks, and that performance in higher-order cognitive tests (planning, episodic memory, sustained attention) would not differ between groups.

## 2 Methods

### 2.1 Participants

80 participants (22 male, 58 female) with a diagnosis of depressive episode according to International Classification of Diseases 10th Revision (ICD-10) criteria were recruited in East of England from primary care and secondary care through local collaborators and NIHR Clinical Research Network. The details of the Insight study protocol were previously published (Khandaker et al., 2018). The diagnosis of depression was confirmed by using Clinical Interview Schedule-Revised (CIS-R) at eligibility. Participants were required to be on the same antidepressant dose for the last four weeks. Total duration of antidepressants and type of antidepressant medication were recorded. None of the participants were on benzodiazepines at the time of the study session. Patients not meeting an ICD-10 diagnosis of depressive disorder were not included. Patients with bipolar disorder, psychotic disorder, and/or ongoing alcohol or drug abuse were excluded. Further exclusion criteria included patients with cancer, current or recurrent infection, HIV, tuberculosis, Hepatitis B or C, rheumatological autoimmune disease, mixed connective tissue disease, scleroderma, polymyositis, or significant systemic involvement secondary to rheumatoid arthritis. All participants completed the same set of cognitive tests and psychiatric measures and gave blood samples to ascertain inflammation status as measured by high-sensitive C-reactive protein (hs-CRP) levels in plasma. Participants were grouped in two according to their hs-CRP levels; those with (hs-CRP ≥ 3 ​mg/L) and those without (hs-CRP < 3 ​mg/L) evidence of low-grade inflammation. CRP ≥3 ​mg/L is a cut-off that is widely used in inflammation studies to represent high levels of inflammation based on the recommendations by the American Heart Association and Centre for Disease Control and Prevention (Pearson et al., 2003). Previous studies (Michal et al., 2013; Köhler-Forsberg et al., 2017) and randomised controlled trials evaluating immunotherapies for depression (Bekhbat et al., 2018; Khandaker et al., 2018) have used this cut-off to reliably identify patients with evidence of low-grade inflammation. All participants also provided blood samples to confirm that they had no infection at the time of participation. The study was approved by the South Central – Oxford B Research Ethics Committee (Reference: 18/SC/0118).

### 2.2 CRP measurement

Blood samples were collected from non-fasting participants, promptly centrifuged and subsequently assayed for serum hs-CRP levels using an automated colorimetric immunoassay on the Siemens Dimension EXL analyser. The minimum detection limit was 0.1 ​mg/L. Acute bacterial infection was excluded by testing white blood cell count. The blood samples were also tested for HIV, Hepatitis B virus, Hepatitis C virus and tuberculosis. All participants had a chest x-ray to exclude any infection. Since the acute inflammation was excluded comprehensively, there was no superior cut-off for hs-CRP values for the participants. All samples were assayed at the Core Biochemical Assay Laboratory, located in Addenbrooke's hospital, Cambridge, by staff blind to psychiatric and cognitive measures.

### 2.3 Psychiatric measures

Participants completed self-administered validated questionnaires for depression, pleasure, and fatigue. Total scores for were calculated by summing individual item scores according to user manuals. For all questionnaires higher scores represent greater symptom severity.

#### 2.3.1 Clinical interview schedule revised (CIS-R)

The CIS-R elicits responses to 14 areas of symptoms including fatigue, appetite, sleep problem, concentration, irritability, depression, depressive ideas, anxiety, worry, panic, phobia, compulsive behaviours, obsessive thoughts and somatic symptoms. It can be used to generate diagnostic categories according to the ICD-10, including diagnosis of depression.

#### 2.3.2 Beck Depression Inventory II (BDI-II)

Depression severity was assessed using Beck Depression Inventory II (Beck et al., 1996)total scores. Each individual item on this 21-item questionnaire was coded on a 4-point scale ranging from 0 to 3 giving a total score of 0–63. BDI-somatic score was also calculated with regards to the relevance to inflammation. This score was calculated by summing seven BDI-II items; 4 ​= ​lack of pleasure, 15 ​= ​loss of energy, 16 ​= ​changes in sleeping pattern, 18 ​= ​changes in appetite, 19 ​= ​concentration difficulty, 20 ​= ​tiredness or fatigue and 21 ​= ​loss of interest in sex.

#### 2.3.3 Multidimensional Fatigue Inventory (MFI)

Fatigue was measured using the Multidimensional Fatigue Inventory (Smets et al., 1995). Responses to each of the items on this 20-item questionnaire were coded on a 5-point scale ranging from 1 to 5 with a total score of 20–100.

#### 2.3.4 Snaith-Hamilton pleasure scale (SHAPS)

The Snaith-Hamilton Pleasure Scale (Snaith et al., 1995) was used to assess symptoms of anhedonia. Each of the 14 items were coded as 0 ​= ​agree and 1 ​= ​disagree with a total score of 0–14.

### 2.4 Cognitive measures

Premorbid IQ was assessed with National Adult Reading Test (Nelson, 1982). The NART assesses participant's vocabulary by presenting them with a list of 50 words with irregular spellings in British English (e.g. “aisle”) and asking them to provide the pronunciation of the words. NART scores are converted to predict IQ scores on the Wechsler Adult Intelligence Scale (Bright et al., 2018).

Cognitive tasks were selected to tap into cognitive domains that are reported to be widely affected in depression. These tests represent potential treatment targets and are well-established in terms of reliability and their sensitivity to pharmacological manipulation.

#### 2.4.1 Lower-order cognition tests

##### 2.4.1.1 CANTAB emotional bias test (EBT)

In this test, participants are asked to rapidly respond to the faces that are morphed between two emotions of varied intensities. We used the happy to sad variant in this study. Each face is displayed for 150 ​ms, followed by a two-alternative forced choice where they must select happy or sad. The key outcome measure for the EBT is the bias point - the proportion of trials selected as happy compared to sad, adjusted to a scale of 0–15. Other key measures are latency periods of responses to each emotion (Cambridge Cognition, 2019).

##### 2.4.1.2 Emotional categorisation and recall test (ECAT)

ECAT has two stages. At first stage (categorisation), the participants are required to respond accordingly when words of changing valence (positive, negative, neutral) are presented rapidly on the screen. At the second stage (recall), the participants are asked to list the words they recall. Key measures are reaction time, accuracy, and the number of words recalled.

##### 2.4.1.3 Symbol coding test

Widely accepted as a psychomotor speed task, symbol coding requires participants to copy symbols corresponding to numbers within 90 ​s. Main measure is the number of correctly coded symbols.

##### 2.4.1.4 CANTAB five choice reaction time test (RTI)

In this test, the participants must respond to the circles on the upper side of the screen from a position of holding a button at the bottom of the screen. This test has two modes (simple and five-choice). In each case, a yellow dot will appear in one of the circles, and the participant must react as soon as possible, releasing the button at the bottom of the screen, and selecting the circle in which the dot appeared. Key measures include reaction time and movement time for both the simple and five-choice modes (Cambridge Cognition, 2019).

#### 2.4.2 Higher order cognition tests

##### 2.4.2.1 CANTAB rapid visual processing (RVP) test

RVP is a test of sustained attention which requires detection of infrequent 3-digit sequences among serially presented digits. Participants are required to detect target sequences of digits (e.g. 2,4,6) among pseudo-randomly appearing set of numbers and to register responses while using the press pad. Test includes a demonstration phase where participants are presented with visual and auditory cues indicating target sequences. Key measures are measures for this task are target sensitivity (RVP A’), median response latency, and probability of false alarms (Cambridge Cognition, 2019).

##### 2.4.2.2 CANTAB one touch stockings of cambridge (OTS) test

OTS is a test of executive function, spatial planning, and working memory. The task interface has different sizes of pockets (stockings) with different coloured balls. Participants are presented two patterns and asked to figure out the minimum number of moves required to match one pattern with another. They must work out in their head how many moves the solutions these problems require, then choose the appropriate box at the bottom of the screen. Outcome measures are median latency to first choice and latency to correct, and problems solved in first choice (Cambridge Cognition, 2019).

##### 2.4.2.3 CANTAB paired associates learning (PAL) test

This test is used to assess visual episodic memory and new learning. Boxes are presented in random order and the participants are asked to detect the pattern. The patterns are then displayed in the middle of the screen, one at a time, and the subject must touch the box where the pattern was originally located. If the subject makes an error, no feedback is given, but the patterns are re-presented to remind the subject of their locations. If a stage is not completed at 10 trials, then test automatically ends, and errors for uncompleted stages are recorded as adjusted values. Key measures are the total number of errors adjusted and first trial memory score (Cambridge Cognition, 2019).

### 2.5 Statistical analysis

**Fig. 1:**
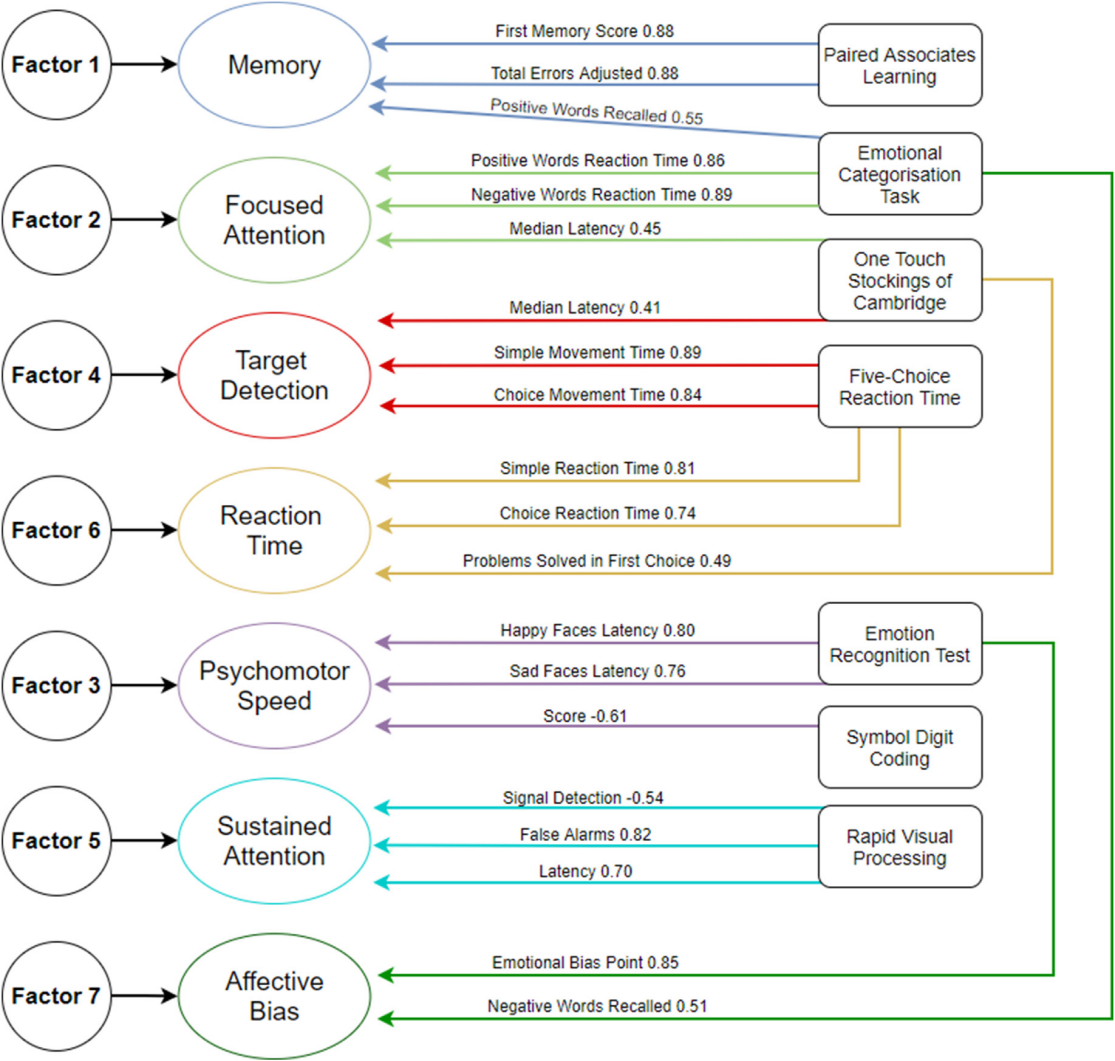
Factor loadings for key neurocognitive measures.

We used key measures from CANTAB tests, number of correct responses for symbol coding test, and number of correctly recalled words and average reaction times for ECAT to assess performance in corresponding domains. All test scores were tested for normal distribution and log transformed before analysis where skewed distribution was observed. Univariate regression using Analysis of Covariance were performed to compare groups with and without evidence of inflammation for each key measure. Sex, age, and body mass index (BMI) were used as covariates. All comparisons were further controlled for depression severity and fatigue scores. For tests that had at least one significant difference between groups, linear regression analyses were used to assess the association between hs-CRP levels and cognitive test performance across the group adjusting for sex, BMI, age, depression severity, and fatigue. Confounders were selected based on previous studies suggesting a link between those factors and inflammation. Higher CRP levels were associated with depression severity (Köhler-Forsberg et al., 2017; Liukkonen et al., 2006) and fatigue (Raison et al., 2009). Further adjustment analyses were run to assess the potential impact of antidepressant type on cognitive functions. We also analysed if certain groups of cognitive functions differ between groups by using factor analysis. We used key measures from each test to yield factor scores by using principal component analysis (Fig. 1) The details of factor analysis are presented in results section. All analyses were conducted with SPSS version 27.

## 3 Results

### 3.1 Baseline characteristics of sample

Clinical and demographic characteristics of depressed patients with and without evidence of inflammation are presented in Table 1. Patients with evidence of inflammation (CRP≥3 ​mg/L) had higher depression severity, higher somatic depressive symptoms, higher BMI, lower percentage of SSRI use and higher fatigue scores. Premorbid IQ, age, percentage of female patients, anhedonia scores and previous depressive episodes were comparable across groups.

**Table 1:** Characteristics of study sample.

Characteristic	Depressed Patients without Evidence of Inflammation (hs- CRP <3 ​mg/L)	Depressed Patients with Evidence of Inflammation (hs- CRP ≥3 ​mg/L)	Test statistic; *P*-value
**Sample, No.**	43	37	–
**Female sex, No. (%)**	72.09%	72.97%	Chi square ​= ​0.008 p ​= ​0.93
**Age, years - Mean (SD)**	36.18 (12.13)	41.14 (11.37)	t ​= ​-1.876 p ​= ​0.06
**Premorbid IQ - Mean (SD)**	118.90 (5.34)	117.59 (5.81)	t ​= ​1.315 p ​= ​0.29
**hsCRP (mg/L) - Mean (SD)**	0.88 (0.72)	8.53 (5.01)	**t ​= ​- 9.197** **p ​< ​0.001**
**BMI - Mean (SD)**	25.65 (6.52)	36.24 (8.19)	**t ​= ​-6.391** **p ​< ​0.001**
**BDI total score - Mean (SD)**	27.58 (9.29)	34.62 (9.08)	**t ​= ​-3.413** **p ​= ​0.001**
**BDI somatic symptom score- Mean (SD)**	9.19 (3.29)	12.16 (3.00)	**t ​= ​-4.198** **p ​< ​0.001**
**Number of previous depressive episodes - Mean (SD)**	3.98 (1.80)	3.97 (1.92)	t ​= ​0.009 p ​= ​0.99
**Duration of antidepressant use (months) - Mean (SD)**	23.35 (38.67)	22.17 (38.38)	t ​= ​0.135 p ​= ​0.89
**SSRI antidepressant users, No (%)**	35 (81.3%)	22 (59.4%)	Chi square ​= ​4.672 p ​= ​0.03
**Multidimensional Fatigue Inventory Score – Mean (SD)**	70.23 (14.05)	83.16 (9.08)	**t ​= ​-4.798** **p ​< ​0.001**
**Snaith-Hamilton Pleasure Scale**	4.23 (3.44)	5.27 (3.57)	t ​= ​-1.321 p ​= ​0.19

Abbreviations - BMI: Body Mass Index, BDI: Beck Depression Inventory.

Categorical variables and proportions were compared using Chi-squared test and continuous variables were compared using independent samples *t*-test.

### 3.2 Comparison of cognitive performance between depressed patients with and without evidence of inflammation

#### 3.2.1 Lower-order cognitive functions

##### 3.2.1.1 Emotional processing

Patients with low hsCRP levels recalled a higher number of positive words and a lower number of negative words in the Emotional Recall Test, but this difference was not statistically significant (Mean difference (positive words recall) ​= ​0.08, 95% CI ​= ​-0.02 – 0.19; negative words recall ​= ​0.06, 95% CI =(-0.05 – 0.18)). Mean differences were smaller after controlling for age, sex, and BMI (Table 2). Further adjustment for depression scores had more impact on the model than other covariates.

**Table 2:** Neurocognitive measures between depressed patients with and without evidence of inflammation.

Measures	Patients without Evidence of Inflammation (n ​= ​43) (CRP<3 ​mg/L) Mean (SD)	Patients wit Evidence of Inflammation (n ​= ​37) (CRP ≥3 ​mg/L) Mean (SD)	Mean Difference (95% CI)^1^					
			Unadjusted	Adjusted for age, sex and BMI	Adjusted for age, sex BMI, and depression	Adjusted for age, sex BMI, and fatigue	Adjusted for age, sex, BMI, depression and fatigue	Test Statistic and p value for final model
CANTAB EBT Bias Point	6.96 (1.44)	7.20 (1.31)	-0.23 (-0.86 – 0.39)	0.13 (-0.75 – 0.77)	0.07 (-0.76 – 0.91)	-0.01 (-0.85 – 0.81)	0.03 (-0.82 – 090)	F ​= ​0.008, p ​= ​0.92
CANTAB EBT Median Reaction Time Sad Faces (miliseconds)	671.80 (163.57)	729.77 (180.41)	-57.96 (-135.64 – 19.70)	-51.05 (-150.04 - 47.94)	-16.26 (-123.26 – 90.74)	-37.36 (-144.822 – 70.10)	-16.21 (-126.78 – 94.35)	F ​= ​0.086, p ​= ​0.77
CANTAB EBT Median Reaction Time Happy Faces (miliseconds)	701.82 (157.44)	737.84 (253.02)	-36.01 (-129.31 – 57.28)	-21.00 (-140.89 – 98.88)	65.03 (-56.82 – 186.90)	13.89 (-114.84 – 142.63)	65.85 (-60.07 – 191.77)	F ​= ​1.088, p ​= ​0.30
ECAT Total Recalled Correct (Positive Words)	3.33 (1.87)	2.76 (1.65)	0.08 (-0.02 – 0.19)	0.06 (-0.06 – 0.20)	0.07 (-0.07 – 0.21)	0.10 (-0.03 – 0.24)	0.09 (-0.05 – 0.23)	F ​= ​1.534, p ​= ​0.54
ECAT Total Recalled Correct (Negative Words)	2.09 (1.55)	2.22 (1.68)	0.06 (-0.05 – 0.18)	0.06 (-0.08 – 0.21)	-0.017 (-0.13 – 0.17)	-0.024 (-0.17 – 0.12)	-0.018 (-0.17 – 0.13)	F ​= ​0.050, p ​= ​0.82
ECAT Accuracy	34.17 (7.89)	34.96 (6.79)	-0.03 (-0.13 – 0.07)	-0.04 (-0.16 – 0.07)	-0.025 (-0.15 – 0.10)	-0.04 (-0.16 – 0.08)	-0.027 (-0.16 – 0.10)	F ​= ​0.160, p ​= ​0.68
ECAT Reaction Time - Positive Words (miliseconds)	998.01 (289.42)	877.08 (346.59)	0.06 (-0.01 – 0.13)	0.05 (-0.028 – 0.14)	0.071 (-0.021 – 0.16)	0.081 (-0.007 – 0.16)	0.082 (-0.01 – 0.17)	F ​= ​3.115, p ​= ​0.08
ECAT Reaction Time - Negative Words (miliseconds)	10007.58 (309.28)	902.34 (300.33)	0.36 (-0.03 – 0.10)	0.03 (-0.05 – 0.11)	0.05 (-0.03 – 0.13)	0.061 (-0.020 – 0.142)	0.061 (-0.024 – 0.146)	F ​= ​2.028, p ​= ​0.15
Digit symbol coding score	70.21 (15.76)	62.14 (17.55)	**∗0.06 (0.003**–**0.11)**	0.036 (-0.030 – 0.10)	0.016 (-0.055 – 0.087)	0.025 (-0.04 – 0.09)	0.014 (-0.059 – 0.087)	F ​= ​0.147, p ​= ​0.70
RTI Median Simple Movement Time (miliseconds)	216.47 (46.82)	243.04 (53.64)	**∗-26.56 (-48.92 - -4.20)**	-24.74 (-52.47 – 2.97)	-25.52 (-55.82 – 4.76)	-22.61 (-52.48 – 7.25)	-23.96 (-55.04 – 7.12)	F ​= ​2.361, p ​= ​0.12
RTI Median Simple Reaction Time (miliseconds)	316.58 (40.77)	320.10 (34.32)	-3.52 (-20.46 - -13.40)	3.59 (-17.25 – 24.45)	11.46 (-10.83 – 33.75)	10.61 (-11.40 – 32.64)	13.95 (-8.77 – 36.68)	F ​= ​1.498, p ​= ​0.22
RTI Median Five-Choice Movement Time (miliseconds)	260.86 (60.21)	281.05 (69.27)	-20.19 (-49.01 – 8.62)	-2.93 (-36.99 – 31.12)	-1.98 (-39.20 - 35.24)	1.34 (-35.28 – 37.97)	0.52 (-37.62 – 38.66)	F ​= ​0.001, p ​= ​0.97
RTI Median Five-Choice Reaction Time (miliseconds)	354.98 (37.44)	375.01 (59.10)	-20.02 (-41.74 – 1.69)	-17.26 (-44.63 – 10.09)	-8.73 (-38.19 – 20.73)	-6.0 (-35.17 – 22.16)	-4.00 (-33.77 – 25.76)	F ​= ​0.072, p ​= ​0.78
RVP A’ (Signal Detection)	0.90 (0.05)	0.89 (0.05)	0.007 (-0.01 – 0.03)	0.009 (-0.02 – 0.03)	0.014 (-0.01 – 0.04)	0.016 (-0.01 – 0.04)	0.017 (-0.015 – 0.04)	F ​= ​1.152, p ​= ​0.28
RVP Median Response Latency (miliseconds)	454.66 (82.03)	448.05 (81.06)	6.60 (-29.81 – 43.03)	37.43 (-4.47 – 79.35)	**∗47.99 (2.62**–**93.35)**	**∗48.05 (3.37**–**92.72)**	**∗52.10 (5.72**–**98.48)**	**F** ​= ​**5.015, p** ​= ​**0.02**
RVP Probability of False Alarm	0.017 (0.05)	0.015 (0.03)	0.002 (-0.01 – 0.02)	0.009 (-0.01 – 0.03)	0.001 (-0.026 – 0.029)	0.002 (-0.026 – 0.029)	-0.001 (-0.03 – 0.02)	F ​= ​0.009, p ​= ​0.92
CANTAB OTS Problems Solved on First Choice	11.00 (2.23)	10.97 (2.65)	-0.15 (-1.26 – 0.96)	0.07 (-1.32 – 1.48)	0.41 (-1.12 – 1.95)	-0.08 (-1.59 – 1.43)	-0.006 (-1.59 – 1.58)	F ​< ​0.001, p ​= ​0.99
CANTAB OTS Median Latency to First Choice (miliseconds)	10517.55 (3708.81)	9750.94 (4190.28)	0.04 (-0.03 – 0.12)	0.06 (-0.02 – 0.15)	0.088 (-0.008 – 0.18)	0.078 (0.01–0.17)	0.09 (-0.01 – 0.19)	F ​= ​3.190, p ​= ​0.07
CANTAB PAL Total Errors Adjusted	10.79 (10.28)	12.30 (12.75)	0.07 (-0.10 – 0.25)	0.006 (-0.20 – 0.21)	-0.08 (-0.31 – 0.14)	-0.07 (-0.30 – 0.15)	-0.10 (-0.33 – 0.13)	F ​= ​0.712, p ​= ​0.40
CANTAB PAL First Attempt Memory Score	14.00 (3.97)	13.19 (3.28)	-0.10 (-0.07 – 0.05)	0.01 (-0.06. – 0.08)	0.06 (-0.02 – 0.15)	0.07 (-0.01 – 0.15)	0.07 (-0.008 – 0.16)	F ​= ​3.263, p ​= ​0.07

Asteriks denote significance level at p ​< ​0.05.

Patients with high hs-CRP had higher bias scores in the CANTAB Emotion Bias Test and slower reaction times to both sad and happy faces while the differences were not statistically significant. Similarly, adjustments for covariates showed that depression severity was the main attenuating factor for Emotion Bias Test performance (Table 2).

##### 3.2.1.2 Processing speed

Patients with high hs-CRP had poorer performance in the symbol coding test (mean difference ​= ​0.06, 95% CI ​= ​0.003–0.11). The difference was not significant after adjusting for age, sex, and BMI. Depression severity was the main attenuating factor (Table 2).

Patients with evidence of inflammation had significantly slower performance in CANTAB RTI Simple Movement Time (mean difference ​= ​26.56, 95% CI ​= ​-48.92 to −4.20). The difference between two groups was no longer significant after controlling for age, sex, and BMI. The group with evidence of inflammation also had slower reaction time in a simple version of CANTAB RTI and slower reaction time and movement time in a five-choice version However, none of these differences were statistically significant (Table 2).

#### 3.2.2 Higher-order cognitive functions

##### 3.2.2.1 Sustained attention

In sustained attention measures (CANTAB RVP), there was no significant difference between groups that remained after adjusting for age, sex, and BMI (Table 2). There was a significant difference in RVP Median Response Latency after additional adjustment for depression and additional adjustment for fatigue (final model F ​= ​5.015, p ​= ​0.02). Interestingly, the effect was based on a slightly longer response latency for the group without evidence of inflammation. Upon close inspection, the strongest predictor for RVP median latency performance was age (F ​= ​18.481, p ​< ​0.001). This result suggests that the link between depression severity, age and latency scores may be stronger for some participants that drove the effect.

##### 3.2.2.2 Memory and planning

Patients in both groups had comparable performance in an episodic memory test (CANTAB PAL) (Table 2).

OTS median latency to first choice was shorter in patients with evidence of inflammation, suggesting that the patient group without evidence of inflammation may be more cautious before making a choice. The difference was not significant and remained so after adjusting for age, sex, and BMI (mean difference ​= ​0.06, 95% CI ​= ​- 0.02–0.15).

#### 3.2.3 Association between CRP levels and neurocognitive performance

Regression analyses for the whole sample showed that RTI Median Simple Movement Time was significantly associated with hs-CRP levels (β ​= ​19.75, 95% CI ​= ​2.55–36.96). The effect was not significant after controlling for age, sex, and BMI, and remained so after further adjustment for depression and fatigue. For other test measures examined, inflammation levels were not found to be associated with neurocognitive test scores (Table 3). It is noteworthy that reaction time was affected more by fatigue scores than depression scores whereas digit symbol coding score was affected more by depression scores (Table 3). Further adjustments for antidepressant type (SSRI versus others) did not show any significant effect of antidepressant type on the association between inflammation and neurocognitive performance.

**Table 3:** Association between CRP levels and Cognitive Test Measures.

Measures	B coefficients (95% CI)					Test Statistic and p value for final model
	Unadjusted	Adjusted for age, sex and BMI	Adjusted for age, sex BMI, and depression	Adjusted for age, sex BMI, and fatigue	Adjusted for age, sex BMI, depression and fatigue	
ECAT Reaction Time - Positive Words (miliseconds)	-0.04 (-0.099 – 0.001), p ​= ​0.054	-0.03 (-0.09 – 0.01)	-0.045 (-0.10 – 0.01)	-0.05 (-0.10 – 0.09)	-0.05 (-0.11 – 0.01)	t ​= ​-1.626, p ​= ​0.10
ECAT Reaction Time - Negative Words (miliseconds)	-0.038 (-0.084 – 0.009), p ​= ​0.11	-0.03 (-0.083 – 0.023)	-0.035 (-0.091 – 0.021)	-0.040 (-0.095 – 0.014)	-0.040 (-0.096 – 0.016)	t ​= ​-1.413, p ​= ​0.16
RTI Median Simple Movement Time (miliseconds)	∗**19.75 (2.55**–**36.96), p** ​= ​**0.02**	18.03 (-1.94 – 38.01)	16.98 (-3.81 – 37.78)	15.61 (-4.98 – 36.20)	17.10 (-4.24 – 38.45)	t ​= ​1.597, p ​= ​0.11

Asteriks denotes statistical significance at p ​< ​0.05 level.

A sensitivity analysis was conducted to investigate the association between cognition and inflammation after excluding the participants with hs-CRP >10 ​mg/L (n ​= ​12). The sensitivity analysis showed that one cognitive measure, RTI Median Simple Movement Time, was significantly associated with CRP levels after adjusting for age, sex, and BMI (t ​= ​2.610, p ​= ​0.01, 95% CI 1.49–11.26). The association remained significant after further adjustment with depression severity and fatigue (t ​= ​2.349, p ​= ​0.02, 95% CI 0.95–11.88).

Further exploratory analyses were conducted to investigate whether cognitive functions were differently affected by somatic symptoms compared to affective symptoms. Analyses showed that there was no significant difference regarding the association of neurocognitive performance and affective and somatic symptoms, with the exception of OTS Median Latency to Correct (MLC). OTS MLC was significantly associated with affective symptoms with a relatively large confidence interval (t ​= ​-2.05, 95% CI ​= ​-42.98 – 0.47, p ​= ​0.045), but not with somatic symptoms (t ​= ​-0.91, 95% CI ​= ​-16.24 – 6.08, p ​= ​0.36).

#### 3.2.4 Factor analysis of cognitive domains

Further analyses were performed using principal component analysis with Varimax rotation and Kaiser Normalization. Using the key cognitive measures for each test, Kaiser-Meyer-Olkin (KMO) Measure of Sampling Adequacy was 0.618. Bartlett's Test of Sphericity yielded approximate Chi Square of ​= ​696.35, (df ​= ​171, significance <0.001). KMO values above 0.6 are required for samples smaller than 100 (MacCallum et al., 1999). Factors with Eigenvalues higher than 1 were retained. Coefficients smaller than 0.3 were suppressed. This analysis yielded a 7-factor structure, explaining 75.31% of the variance (Table 4). The 7 factors included memory, emotional bias, focused attention, target detection, psychomotor speed, sustained attention, and reaction time. Factor loadings of key cognitive measures are presented in Fig. 1. We named the factors as follows: F1 – memory, F2 – focused attention, F3 – psychomotor speed, F4 – target detection, F5 – sustained attention, F6 – reaction time, and F7 – affective bias. Additionally, all neurocognitive measures were forced into a single factor yielding a component score. The single factor explained 25.65% of the variance. Regression factor scores were then analysed to assess their association with inflammation. None of the factors were associated with CRP levels (Table 5). Factor 3 (psychomotor speed) and factor 5 (sustained attention) were more sensitive to adjustment for depression scores.

**Table 4:** Factor loadings for cognitive measures.

Rotated Component Matrix							
	Component						
	1	2	3	4	5	6	7
CANTAB PAL First Attempt Memory Score	.886						
CANTAB PAL Total Errors Adjusted	-.884						
ECAT Total Recalled Correct (Positive Words)	.551						
ECAT Reaction Time - Negative Words		.892					
ECAT Reaction Time - Positive Words		.865					
CANTAB OTS Median Latency to First Choice		.450		.416			
CANTAB EBT Median Reaction Time Happy Faces			.800				
CANTAB EBT Median Reaction Time Sad Faces			.760				
Digit symbol coding score			-.614				
RTI Median Simple Movement Time				.897			
RTI Median Five-Choice Movement Time				.847			
RVP Probability of False Alarm					.828		
RVP Median Response Latency					.703		
RVP A’ (Signal Detection)					-.548		
RTI Median Simple Reaction Time						.812	
RTI Median Five-Choice Reaction Time						.745	
CANTAB OTS Problems Solved in First Choice						.494	
CANTAB EBT Bias Point							.857
ECAT Total Recalled Correct (Negative Words)							.519

Kaiser-Meyer-Olkin Measure of Sampling Adequacy ​= ​0.618. Bartlett's Test of Sphericity – Approx. Chi Square ​= ​696.35, df ​= ​171, significance <0.001. Extraction Method: Principal Component Analysis. Rotation Method: Varimax with Kaiser Normalization.

Rotation converged in 10 iterations. Loadings above 0.4 absolute values are presented. 7 Factors explained 75.31% of the variance. When all items are forced into one factor, one factor explained 25.65% of the variance.

**Table 5:** Associations of CRP level with factor scores for cognitive measures.

Factors	B coefficients (95% CI)					Test Statistic and p value for final model
	Unadjusted	Adjusted for age, sex and BMI	Adjusted for age, sex BMI, and depression	Adjusted for age, sex BMI, and fatigue	Adjusted for age, sex BMI, depression and fatigue	
Factor 1 (Memory)	0.05 (-0.30 – 0.40)	-0.031 (-0.43 – 0.37)	-0.061 (-0.49 – 0.37)	-0.069 (-0.49 – 0.35)	-0.076 (-0.51 – 0.36)	t ​= ​-0.346, p ​= ​0.73
Factor 2 (Focused Attention)	-0.32 (-0.67 – 0.01)	-0.23 (-0.61 – 0.14)	-0.25 (-0.65 – 0.14)	-0.27 (-0.67 – 0.11)	-0.27 (-0.68 – 0.12)	t ​= ​-1.363, p ​= ​0.17
Factor 3 (Psychomotor Speed)	0.15 (-0.19 – 0.50)	0.04 (-0.37 – 0.46)	-0.12 (-0.54 – 0.30)	-0.039 (-0.47 – 0.39)	-0.13 (-0.56 – 0.30)	t ​= ​-0.602, p ​= ​0.54
Factor 4 (Target Detection)	0.25 (-0.08 – 0.60)	0.24 (-0.15 – 0.63)	0.24 (-0.17 – 0.65)	0.24 (-0.16 – 0.65)	0.24 (-0.18 – 0.66)	t ​= ​1.141, p ​= ​0.25
Factor 5 (Sustained Attention)	-0.12 (-0.47 – 0.22)	-0.21 (-0.63 – 0.20)	-0.15 (-0.59 – 0.28)	-0.18 (-0.61 – 0.52)	-0.15 (-0.60 – 0.29)	t ​= ​-0.689, p ​= ​0.49
Factor 6 (Reaction Time)	0.12 (-0.22 – 0.47)	0.03 (-0.38 – 0.44)	-0.07 (-0.50 – 0.35)	-0.04 (-0.46 – 0.39)	-0.09 (-0.53 – 0.34)	t ​= ​-0.424, p ​= ​0.67
Factor 7 (Emotional Bias)	0.008 (-0.34 – 0.36)	-0.13 (-0.54 – 0.27)	-0.137 (-0.56 – 0.29)	-0.11 (-0.53 – 0.31)	-0.12 (-0.56 – 0.31)	t ​= ​-0.559, p ​= ​0.57
Single Factor	0.030 (-0.32 – 0.38)	-0.007 (-0.38 – 0.38)	-0.10 (-0.50 – 0.30)	-0.06 (-0.46 – 0.33)	-0.11 (-0.52 – 0.29)	t ​= ​-0.556, p ​= ​0.58

## 4 Discussion

### 4.1 Findings

In this study sample of 80 patients with depression and somatic symptoms, we investigated the link between inflammation and cognition. Patients with evidence of inflammation (hs-CRP ≥3 ​mg/L) showed poorer performance in processing speed and reaction time compared to those without evidence of inflammation (hs-CRP <3 ​mg/L). The effect was no longer significant after controlling for sex, BMI and age and remained so after further adjustments for depression and fatigue scores. Overall, there was a pattern in findings that cognitive outcomes measuring response timings or lower-order cognition were influenced more by adjustment of fatigue scores. On the other hand, cognitive outcomes that are linked to higher-order cognitive functions (i.e. memory, planning, executive processes) were affected more by adjustment of depression scores. Our initial hypothesis was that higher CRP would affect low-level cognitive functions but the impact on higher cognitive functions would be indirect. Across the whole group, there was no significant association between CRP levels and the cognitive domains assessed. However, there were indications that different cognitive processes were more sensitive to certain predictors than others (Table 3). Cognitive domains elicited by factor analysis of our own sample showed similar trends in the distinction of lower-order and higher-order cognitive functions.

We reported results of various cognitive domains and possible links with inflammation from a sample of depressed patients. Our results suggested that some cognitive domains may be more susceptible to alterations in inflammatory status. There is limited data regarding the impact of inflammation on cognitive domains. The interaction between inflammation and neuropsychological mechanisms in depression is yet to be elucidated. We believe that integration of findings from inflammation and neurocognitive research in depression will help our understanding of the heterogeneous factors at play regarding cognitive dysfunction in depression. Such integration will be helpful to identify better treatment targets. The cognitive neuropsychological model of depression can provide insights into how lower-order cognitive systems and higher-order systems operate and interact (Roiser et al., 2012).

### 4.2 Links between inflammation and cognition in depression

Previously, few studies reported specific analyses regarding CRP and cognitive functions. There are several discrepancies in findings, specifically methodological differences and sample characteristics. One study reported that higher CRP levels were associated with lower psychomotor speed performance as measured by Trail Making Test (Krogh et al., 2014). In terms of sample characteristics, patients in Krogh et al. (2014) study had relatively low CRP levels and less severe depressive symptoms compared to our sample. It may be possible that the impact of CRP on cognition might have been less prominent when patients are more severely affected by depression. Another important factor in discrepancy among studies is the cognitive tests used. In a study (Goldsmith et al., 2016) that used at least two of the measures in our study (namely CANTAB RTI and Symbol Coding), no association between CRP and psychomotor speed was found. They instead reported an inverse association between IL-6 levels and psychomotor speed (Goldsmith et al., 2016) and suggested that cytokines may be more sensitive inflammatory markers regarding changes in cognitive function. The rapid changing nature of cytokine measures should also be taken into consideration as cytokine levels on the day of the study may be affected by stress levels. In a meta-analysis, IL-6 and IL-1β were the most rapidly changing inflammatory markers under stress while the change in CRP levels was not significant (Steptoe et al., 2007). Considering the possible stressful impact of cognitive test sessions, these results suggest that CRP levels may be temporally more stable marker to help identify links between inflammation and cognition. It should be noted that some other studies suggested a cut-off of CRP>1 ​mg/L (Fond et al., 2021; Liukkonen et al., 2006). In this study, we used the widely accepted CRP cut-off of 3 ​mg/L as evidence of inflammation in line with previous studies (Michal et al., 2013; Köhler-Forsberg et al., 2017; Foley et al., 2021).

Reaction time can be considered as a cognitive skill that is immediately linked with the bodily changes associated with inflammation. Evidence from experimentally induced inflammation via typhoid vaccine (Brydon et al., 2008) suggests that vaccinated participants have drastic slowing in reaction time. Treatment with pro-inflammatory medication (Majer et al., 2008) interferon-gamma was associated with increased reaction time in the CANTAB RTI task. There was no significant effect of interferon treatment on an executive functions task (CANTAB ID/ED – a measure of set-shifting). Cognitive functions were strongly correlated with fatigue further supporting the proposition that reaction time is relatively more in line with the systemic changes. In parallel, Chang et al. (2012) reported no association between set-shifting performance and CRP levels whereas poorer performance in psychomotor speed tasks was associated with high CRP levels in the same study. It should be noted that some cognitive measures span several cognitive domains. Digit symbol coding is regarded as a psychomotor speed test but also requires executive processes and planning abilities. Episodic memory is a key cognitive domain and could be regarded as a higher-order cognitive function. Yet, neural circuitry mediating episodic memory (including hippocampal areas) is sensitive to effects of stress, and therefore is relatively vulnerable to systemic inflammation effects. In our study, episodic memory and emotional verbal recall scores were not associated with hs-CRP levels. In a study of female patients with recurrent depression, Grassi-Oliveira et al. (2011) reported an association between IL-6 and poorer verbal recall performance.

The cognitive neuropsychological model of depression is a helpful framework to understand the upstream and downstream effects of various factors that are involved in development and maintenance of depressive mood (Roiser et al., 2012). So far, it has been unclear how inflammation related changes in mood can be incorporated into the model. Chronic exposure to low-grade inflammation is considered to induce changes in neurotransmitter systems that are linked with depressive symptoms. Treatment with interferon-alpha and associated increase in cytokine levels were shown to activate a brain inflammation response that interacted with serotonin metabolism (Raison et al., 2009). According to the cognitive neuropsychological model, stressful experiences may induce a bottom-up stream of compromised monoamine transmission leading to changes in brain circuits mediating affective processing (Roiser et al., 2012). In our sample, all patients were on a stable dose of antidepressant and the period of use was comparable between patients with and without evidence of inflammation. Therefore, the differences between groups were unlikely to be attributed to monoaminergic transmission.

A key remaining question is to what extent systemic changes directly impact inflammation in the brain. A recent study reported that peripheral CRP was correlated with CRP levels in cerebrospinal fluid (CSF) (Felger et al., 2020). Patients with high peripheral CRP levels had higher CSF cytokine levels which in turn was associated with reduced motivation and anhedonia. On the other hand, measuring inflammation in the brain proved challenging. Currently available measures such as translocator protein binding, are regarded rather indirect indicators of central inflammation, possibly related to special immune mechanisms at play in the brain (Holmes et al., 2018). Longitudinal studies are needed to establish the concordance between peripheral and central measures of inflammation. We should also consider the complexity of the inflammation processes and be mindful of the limitations of the markers. Pathophysiology of depression is multilayered and the alterations in inflammatory markers can be regarded as manifestations of the underlying complex processes. A critical review of the literature highlighted the role of circulating CRP in adaptive repair processes (Del Giudice and Gangestad, 2018). To what extent increased inflammation is adaptive, when those responses become maladaptive, and how these are linked to depression are questions that warrant a multidisciplinary approach including an evolutionary perspective (Raison and Miller 2013).

Our results pointed out certain trends suggesting links between inflammation and certain cognitive functions. We need larger samples and longitudinal studies to examine the links more in detail. Previously, Chang et al. (2012) showed an association between persistence of cognitive dysfunction at 6 weeks of treatment and higher baseline CRP levels. Krogh et al. (2014) reported more benefits from an exercise intervention in patients with lower CRP levels at baseline. Cognitive dysfunction is not solely linked to symptomatic improvement as significant proportion of patients continue to report cognitive problems even after months of symptomatic remission (Conradi et al., 2011). Future studies should investigate the link between inflammation status and cognition in patients longitudinally to understand the contribution of inflammation to persistent cognitive dysfunction in remitted patients. Comprehensive studies with larger samples including analysis of other inflammatory markers alongside a range of cognitive domains are warranted.

### 4.3 Limitations

The main limitation of the study was the relatively small sample size. This may have led to small effects disappearing after controlling for confounders. Since the results from this study are cross-sectional, the associations are limited to the session. Lack of healthy control group meant that it was not possible to compare the findings to a non-clinical population. We used hs-CRP as the index of inflammation that may be subject to confounders. Being limited to CRP, this study lacks the other inflammatory marker measures.

## 4.4 Conclusions

This study showed that depressed patients with evidence of inflammation and somatic symptoms had poorer performance in certain cognitive domains particularly reaction time and psychomotor processing, as compared to depressed patients without evidence of inflammation. However, these effects were fully explained by age, sex, and BMI. Patients in both groups had comparable performance in higher-order cognition tests. Findings from this small exploratory study suggest that stratification of clinical samples according to inflammation status may provide insights into how cognitive functions are affected in depression. In future, studies of similar comprehensive and reliable cognitive measures alongside a range of inflammatory markers are needed to understand the links between inflammation and cognition in depressed patients. Longitudinal studies with larger samples are required to elucidate the role of inflammation in cognitive dysfunction in depression.

## Role of the funding source

This work was funded by a 10.13039/100010269Wellcome Trust fellowship to GMK (grant code: 201486/Z/16/Z). MK was supported by an 10.13039/501100000272NIHR Clinical Lectureship. GMK also acknowledges funding support from Cambridgeshire and Peterborough NHS Foundation Trust R&D Department (Grant code: G101481), the 10.13039/501100011950BMA Foundation (J Moulton grant 2019); the 10.13039/100011705MQ: Transforming Mental Health (grant code: MQDS17/40); and the 10.13039/501100000265Medical Research Council UK (grant codes: MC_PC_17213, MR/S037675/1, and MR/W014416/1). The 10.13039/501100011950BMA Foundation J Moulton grant also supported ÉMF. The funding sources had no role in study design; collection, analysis, and interpretation of data; writing of the report; and the decision to submit the paper for publication.

## Declaration of competing interest

The authors would like to acknowledge the information below and report that they have no conflict of interest regarding the manuscript entitled “Neurocognitive Performance in Depressed Patients with Low-grade Inflammation and Somatic Symptoms”.
